# Effectiveness and safety of intravenous golimumab with and without concomitant methotrexate in patients with rheumatoid arthritis in the prospective, noninterventional AWARE study

**DOI:** 10.1186/s41927-023-00329-8

**Published:** 2023-03-27

**Authors:** Aaron Broadwell, Joy Schechtman, Douglas Conaway, Alan Kivitz, Natalie J. Shiff, Shawn Black, Stephen Xu, Wayne Langholff, Sergio Schwartzman, Jeffrey R. Curtis

**Affiliations:** 1 Rheumatology and Osteoporosis Specialists, 820 Jordan Street, Suite 201, Shreveport, LA 71101 USA; 2 SunValley Arthritis Center, Peoria, AZ USA; 3 Carolina Health Specialists, Myrtle Beach, SC USA; 4 Altoona Center for Clinical Research, Duncansville, PA USA; 5 Janssen Scientific Affairs, LLC, Horsham, PA USA; 6 Adjunct, Community Health and Epidemiology, University of Saskatchewan, Saskatoon, Canada; 7 Janssen Research and Development, LLC, Spring House, PA USA; 8 Weill Cornell Medical College, New York, NY USA; 9 University of Alabama at Birmingham, Birmingham, AL USA

**Keywords:** Intravenous golimumab, Methotrexate, Clinical disease activity index, Rheumatoid arthritis, Safety, Infusion reactions

## Abstract

**Background:**

Biologic therapies are often prescribed for patients with rheumatoid arthritis (RA) who have inadequate responses to or are intolerant of methotrexate (MTX) and patients with poor prognostic indicators. This post hoc analysis evaluated effectiveness and safety of intravenous golimumab + MTX vs golimumab without MTX in RA patients.

**Methods:**

AWARE, a real-world, prospective and pragmatic, Phase 4 study, compared effectiveness and safety of golimumab and infliximab in biologic-naïve and biologic-experienced patients. All treatment decisions were at the discretion of the treating rheumatologist. Effectiveness was evaluated by mean change in CDAI scores at Months 6 and 12. Safety was monitored through approximately 1 year.

**Results:**

Among 685 golimumab-treated patients, 420 (61%) received concomitant MTX during the study and 265 (39%) did not receive MTX after enrollment; 63% and 72%, respectively, discontinued the study. Relative to golimumab without MTX, golimumab + MTX patients had shorter mean disease duration (8.7 vs 10.0 years) and a lower proportion received prior biologics (60% vs 72%); mean ± standard deviation (SD) baseline CDAI scores were similar (30.8 ± 15.1 and 32.6 ± 15.4). Mean ± SD changes from baseline in CDAI scores at Months 6 and 12, respectively, were similar with golimumab + MTX (− 10.2 ± 14.2 and − 10.8 ± 13.8) and golimumab without MTX (− 9.6 ± 12.9 and − 9.9 ± 13.1). The incidence of adverse events/100 patient-years (PY) (95% confidence interval [CI]) was 155.6 (145.6, 166.1) for golimumab + MTX and 191.2 (176.2, 207.1) for golimumab without MTX; infections were the most common type. The incidence of infusion reactions/100PY (95% CI) was 2.1 (1.1, 3.6) for golimumab + MTX versus 5.1 (2.9, 8.3) for golimumab without MTX; none were considered serious. For golimumab + MTX versus golimumab without MTX, rates/100PY (95% CI) of serious infections, opportunistic infections, and malignancies were 2.6 (1.5, 4.3) versus 7.0 (4.4, 10.6), 0.9 (0.3, 2.0) versus 2.6 (1.1, 5.0), and 3.0 (1.7, 4.7) versus 1.0 (0.2, 2.8), respectively.

**Conclusions:**

Mean change in CDAI score in the  golimumab without MTX group was generally similar to that of the golimumab + MTX group through 1 year, regardless of prior biologic therapy. Adverse events were consistent with the known IV golimumab safety profile. These results provide real world evidential data that may assist healthcare providers and patients with RA in making informed treatment decisions.

*Trial registration*: clinicaltrials.gov NCT02728934 05/04/2016.

**Supplementary Information:**

The online version contains supplementary material available at 10.1186/s41927-023-00329-8.

## Background

Rheumatoid arthritis (RA) is a chronic, systemic, inflammatory disease characterized by symmetrical joint synovitis of the hands and feet [1]. Extra-articular disorders such as ischemic heart disease, interstitial lung disease, pericarditis, pleural effusion, or bronchiectasis, are also common in patients with RA, even in those with only minimal articular involvement [1]. Furthermore, patients with RA are at greater risk for comorbidities across many organ systems (e.g., serious infection, osteoporosis, and malignancy) as well as a higher mortality rate than the general population [1].

In the mid-1980s, the conventional synthetic disease-modifying anti-rheumatic drug (csDMARD) methotrexate (MTX) emerged as the standard-of-care for RA patients [2]. Current guidelines from the American College of Rheumatology (ACR) and European Alliance of Associations for Rheumatology (EULAR) continue to recommend MTX as first-line therapy for DMARD-naïve patients with moderate-to-high disease activity [3, 4]. Escalation of therapy with the use of biologics, such as tumor necrosis factor inhibitors (TNFi), is generally recommended for patients with active disease despite MTX or for patients presenting with poor prognostic factors [3, 4]. TNFi have consistently demonstrated significantly greater efficacy in improving the signs and symptoms of RA, physical function, and health-related quality of life, either as monotherapy or in combination with MTX, compared with MTX alone [5–10]. Furthermore, TNFi can offer greater protection against radiographic damage compared with csDMARDs (e.g., MTX, leflunomide, and sulfasalazine) [11].

In some patients, significant toxicities attributed to MTX, including gastrointestinal adverse events (AEs), decreased blood cell counts, and abnormal liver function tests [2, 12], can lead to poor adherence. In addition, women of child-bearing age may be reluctant to use MTX, and healthcare providers may be reluctant to prescribe MTX to these patients due to the known teratogenic effects [13]. In a real-world study, approximately one-third of RA patients treated with a TNFi experienced dose reduction/discontinuation of concomitant MTX secondary to intolerance or AEs over a 2-year follow-up period [14]. Results from a previous observational study of MTX use in patients with RA, found that 26% reported nonadherence during the first 6 months of treatment, with 71% of those patients indicating that the nonadherence was intentional [15]. Among patients who provided a reason for nonadherence, 34% reported this was due to adverse effects of MTX, including nausea, headache, fatigue, and dizziness [15]. Many patients experience significant short-term side effects (e.g., gastrointestinal symptoms, fatigue, malaise) that are temporally related to MTX use, often occurring 1–2 days after weekly dosing, which may be extremely bothersome [16]. In an earlier literature review, Curtis et al. [17] found that adherence to MTX among patients with RA was highly variable. They also noted that persistence with MTX varied from 50 to 94% at 1 year and from 25 to 79% at 5 years, with intolerance being the most common reason for discontinuation.

The TNFi intravenous (IV) golimumab and infliximab are approved to treat adults with RA in combination with MTX [18, 19]. In the prospective and pragmatic, noninterventional, Phase 4 AWARE study, the safety and effectiveness of golimumab and infliximab were evaluated in patients with RA in a real-world setting [20]. Approximately 40% of patients in the AWARE study reported no concomitant MTX use; thus, exploratory, post hoc analyses were undertaken to further evaluate the effectiveness and safety of golimumab with and without concomitant MTX in RA patients.

## Methods

### Patients and study design

AWARE (NCT02728934) was an observational, prospective, noninterventional, real-world study conducted at 88 sites in the United States (US). Details of the patient inclusion criteria and the study design of AWARE have been previously reported [20]. Briefly, adults (≥ 18 years) with a confirmed diagnosis of RA were eligible for enrollment if they were initiating therapy with either IV golimumab or infliximab. Prior biologic therapy was permitted, with the exception of golimumab if enrolling in the golimumab group and infliximab if enrolling in the infliximab group. After enrollment, patients could switch from golimumab to infliximab (or infliximab biosimilar) or from infliximab to golimumab, provided they had not had previous exposure to the second therapy.

No study drug was provided by the sponsor, and all treatment decisions, including prescribed dose and dosing intervals of golimumab and infliximab as well as concomitant medications for RA or other conditions, were at the discretion of the treating rheumatologist. In the US, golimumab is approved at a dosage of 2 mg/kg (over 30 min) at Weeks 0 and 4, and every 8 weeks thereafter [18], and infliximab is approved at a dosage of 3 mg/kg (over 120 min) at Weeks 0, 2, and 6, and every 8 weeks thereafter with dose adjustments up to 10 mg/kg with administration as often as every 4 weeks [19]. According to the Food and Drug Administration (FDA)-approved indication for RA, both golimumab and infliximab are approved for use with concomitant MTX.

AWARE was planned as a 3-year study but was closed after 2 years when sufficient numbers of patients had been treated through Week 52 and it was determined that the primary and major secondary endpoints were achieved.

This study was conducted according to the Declaration of Helsinki and the International Committee on Harmonisation good clinical practices. The study protocol was reviewed by a centralized institutional review board (Copernicus Group, Approval QUI1-15-645); ethics committee review was not performed as there were no study sites outside the US. All patients provided written informed consent.

### Assessments

Patient visits (and corresponding data collection) were completed according to usual clinical practice and could differ  across study sites. The Clinical Disease Activity Index (CDAI) [21] score was determined at baseline, Month 3, Month 6, and Month 12 to evaluate treatment effectiveness (remission [score ≤ 2.8], low disease activity [score > 2.8 to ≤ 10], moderate disease activity [score > 10 to ≤ 22], and high disease activity [score > 22]). Safety was assessed throughout the study. Infusion reactions were defined as any AE that occurred during an infusion or within 1 h after the infusion. AEs of special interest included serious infections, latent tuberculosis, opportunistic infections, and malignancies.

### Statistical methods

In the post hoc analyses reported here, only data from patients who received golimumab were included; results are summarized by concomitant MTX use (i.e., with and without MTX) and prior biologic use (biologic-naïve vs biologic-experienced). Concomitant MTX use was defined as ≥ 1 administration at any time either at or post-Week 0. The use of MTX, including dosage, was not prespecified in the protocol and was at the discretion of the investigator. The primary endpoint (proportion of patients having an infusion reaction) and major secondary endpoints (mean change in CDAI at Months 6 and 12 in biologic-naïve patients) were assessed in the formal, preplanned, interim analysis conducted after approximately 66% of the patients completed Week 52 or permanently discontinued the study [20].

Baseline demographic and disease characteristics, prior biologic use, prior or concomitant therapy with csDMARDs and targeted synthetic (ts)DMARDs, smoking status, and selected comorbid conditions are summarized using descriptive statistics (counts and percentages; means and standard deviations [SDs]). Descriptive statistics are also reported for outcomes through the Week 52 database lock (February 1, 2019). Mean changes from baseline to Months 6 and 12 in CDAI score were determined using both imputed and observed data; all golimumab-treated patients with ≥ 1 post-baseline CDAI score were included in the analyses. In the analysis using imputation for missing data, missing CDAI scores at baseline were imputed using the mean score derived from non-missing baseline values, missing CDAI scores post-baseline were imputed utilizing last observed carried forward (LOCF), and for patients who discontinued due to lack of effectiveness (per the reason provided by the patient and/or the investigator), a change of 0 in CDAI was imputed for subsequent timepoints. In addition, inverse probability of treatment weighted (IPTW) propensity scores were utilized in the analysis of covariance model of change in CDAI to adjust for baseline characteristics of the golimumab + MTX and golimumab without MTX groups. Propensity scores were estimated using logistic regression analysis with concomitant MTX use as the dependent variable. The propensity score model included the following baseline covariates: age, sex, White race (yes/no), US geographic region (East/West), body mass index, weight, disease duration, CDAI score, biologic-naïve (yes/no), other medications (excluding MTX), number of prior biologics received, prior TNFi therapy (yes/no), selected comorbidities (diabetes mellitus, malignancy, cardiovascular, hyperlipidemia, psychiatric history), and smoking status [20]. The least square (LS) mean difference and 95% confidence interval (CI) were determined via analysis of covariance controlling for baseline CDAI. In addition, the proportions of patients in each CDAI disease activity category (remission, low disease activity, moderate disease activity, and high disease activity) at baseline and Months 3, 6, and 12 are summarized by treatment group and prior biologic use (all patients, biologic-naïve, and biologic-experienced).

AEs, serious AEs (SAEs), and AEs of special interest were summarized as counts, percentages, and number of events per 100 patient-years (PY) to adjust for variability in treatment exposure.

## Results

### Baseline characteristics and patient disposition

A total of 1270 patients were enrolled in the AWARE study; 685 received golimumab, and 585 received infliximab. Twenty-three (1.8%) patients switched therapies: 11 who initially received golimumab switched to infliximab and 12 switched from infliximab to golimumab. In addition, 4 patients switched from infliximab to a biosimilar infliximab.

Patient characteristics for the full study population have been previously detailed [20]. Among the 685 golimumab-treated patients, 420 (61%) reported receiving ≥ 1 dose of MTX during the study (golimumab + MTX group) and 265 (39%) reported no MTX use through the Week 52 database lock (golimumab without MTX group). Among the 420 patients categorized as receiving MTX, 3.8% reported concomitant MTX use during the study  while 96.2% reported prior and concomitant use. Prior MTX use was reported by 111 (42%) patients in the golimumab without MTX group.

Baseline characteristics were generally similar between patients in the golimumab + MTX and golimumab without MTX groups, with the exceptions that the former had a numerically higher proportion of females (87% vs 81%, respectively) and a shorter mean ± SD disease duration (8.7 ± 9.4 vs 10.0 ± 10.8 years, respectively) (Table 1). Mean ± SD baseline CDAI scores in the golimumab + MTX and golimumab without MTX groups indicated highly active disease (30.8 ± 15.1 and 32.6 ± 15.4, respectively); mean scores were similar for biologic-naïve and biologic-experienced patients regardless of concomitant MTX usage.

**Table 1:** Baseline demographic and disease characteristics, comorbidities, and prior and concomitant medications for RA

	IV golimumab + methotrexate^1^	IV golimumab without methotrexate	Total
Patients, N	420	265	685
Age, years (range)	61.3 ± 12.8 (21, 88)	60.3 ± 14.4 (22, 89)	60.9 ± 13.4 (21, 89)
Female	367 (87.4)	215 (81.1)	582 (85.0)
Weight, kg, N	397	251	648
	82.6 ± 23.5	83.7 ± 22.0	83.0 ± 22.9
BMI, kg/m^2^, N	392	249	641
All patients	30.7 ± 8.1	30.5 ± 7.7	30.6 ± 8.0
Underweight (< 18.5)	7 (1.8)	4 (1.6)	11 (1.7)
Normal (18.5 to < 25)	96 (24.5)	57 (22.9)	153 (23.9)
Overweight (25 to < 30)	106 (27.0)	72 (28.9)	178 (27.8)
Obese (≥ 30)	183 (46.7)	116 (46.6)	299 (46.6)
Smoking status			
Current	42 (10.0)	25 (9.4)	67 (9.8)
Former	84 (20.0)	71 (26.8)	155 (22.6)
Never	133 (31.7)	77 (29.1)	210 (30.7)
Unknown	161 (38.3)	92 (34.7)	253 (36.9)
Disease duration, years	8.7 ± 9.4	10.0 ± 10.8	9.2 ± 10.0
CDAI score (0–76)^2^			
All patients, N	419	263	682
	30.8 ± 15.1	32.6 ± 15.4	31.5 ± 15.2
Biologic-naïve patients, n	168	73	241
	29.1 ± 14.1	31.3 ± 16.0	29.8 ± 14.7
Biologic-experienced patients, n	251	190	441
	32.0 ± 15.6	33.1 ± 15.2	32.4 ± 15.4
Comorbidities^3^			
Myocardial infarction	7 (1.7)	3 (1.1)	10 (1.5)
Congestive heart failure	4 (1.0)	6 (2.3)	10 (1.5)
Peripheral vascular disease	9 (2.1)	5 (1.9)	14 (2.0)
Cerebrovascular disease	10 (2.4)	5 (1.9)	15 (2.2)
Diabetes mellitus	62 (14.8)	38 (14.3)	100 (14.6)
Malignancies	17 (4.0)	11 (4.2)	28 (4.1)
Liver disease	4 (1.0)	2 (0.8)	6 (0.9)
Hyperlipidemia	113 (26.9)	78 (29.4)	191 (27.9)
Prior biologic treatment	252 (60.0)	191 (72.1)	443 (64.7)
1 biologic	125 (29.8)	73 (27.5)	198 (28.9)
2 biologics	58 (13.8)	49 (18.5)	107 (15.6)
3+ biologics	69 (16.4)	69 (26.0)	138 (20.1)
Prior and concomitant RA medications			
Any prior or concomitant csDMARDs	420 (100)	160 (60.4)	580 (84.7)
csDMARDs other than MTX^4^	118 (28.1)	129 (48.7)	247 (36.1)
1 csDMARDs	302 (71.9)	70 (26.4)	372 (54.3)
≥ 2 csDMARDs	118 (28.1)	90 (34.0)	208 (30.4)
Any prior or concomitant tsDMARDS^5^	26 (6.2)	26 (9.8)	52 (7.6)

Data presented as n (%) or mean ± standard deviation, unless otherwise specified

*BMI* body mass index, *CDAI* Clinical Disease Activity Index, *cs/tsDMARD* conventional synthetic/targeted synthetic disease-modifying anti-rheumatic drug, *IV* intravenous, *RA* rheumatoid arthritis

^1^Includes patients who reported using concomitant methotrexate either at baseline or at any time during the study

^2^Higher scores indicate more severe disease

^3^History of prior or ongoing comorbidity at study entry

^4^Includes azathioprine, gold (sodium aurothiomalate or auranofin), hydroxychloroquine, leflunomide, mycophenolate, and sulfasalazine

^5^Includes baricitinib and tofacitinib

The proportions of patients reporting select prior or ongoing comorbidities were similar between the golimumab + MTX and golimumab without MTX groups Hyperlipidemia (27.9%) and diabetes mellitus (14.6%) were reported most often, while myocardial infarction (1.5%) and congestive heart failure (1.5%) were reported infrequently (Table 1).

At baseline, a lower proportion of patients in the golimumab + MTX group (60%) than in the golimumab without MTX group (72%) reported prior biologic use. Exposure to csDMARDs and tsDMARDs either prior to or during the study is summarized in Table 1. Prior/concomitant use of csDMARDs other than MTX was more common in the golimumab without MTX group (49%) than in the golimumab + MTX group (28%); patients in the golimumab without MTX group also more commonly reported having ever received ≥ 2 csDMARDs (34% vs 28%, respectively) (Table 1). Use of tsDMARDs was less common; 6% patients in the golimumab + MTX group and 10% of those in the golimumab without MTX group reported ever using tofacitinib and one patient (golimumab + MTX group) reported ever receiving baricitinib. In the golimumab + MTX group, the use of ≥ 2 csDMARDs was more common among biologic-experienced patients (30%) than in biologic-naïve (25%) patients; however, the opposite trend was observed in the golimumab without MTX group (biologic-experienced: 32%; biologic-naïve: 38%). Biologic-experienced patients more frequently reported ever receiving tofacitinib compared with biologic-naïve patients in both the golimumab + MTX (9% vs 1%, respectively) and golimumab without MTX (12% vs 4%, respectively) groups.

Overall, 454 (66%) golimumab patients discontinued the study prior to the Week-52 database lock (February 1, 2019): 263/420 (63%) in the golimumab + MTX group and 191/265 (72%) in the golimumab without MTX group. The primary reasons for study discontinuation included lack of effectiveness (27.1% for golimumab + MTX and 32.1% for golimumab without MTX) and AEs (9.3% for golimumab + MTX and 12.5% for golimumab without MTX). A total of 613 patients had missing data that required data imputation (LOCF).

### Clinical effectiveness

As previously reported in the formal interim analysis of biologic-naïve patients, the mean changes from baseline in CDAI at 6 and 12 months in the golimumab group were − 9.5 and − 9.4, respectively, and were noninferior to those in infliximab patients (− 10.1 and − 10.1, respectively) [20].

**Fig. 1:**
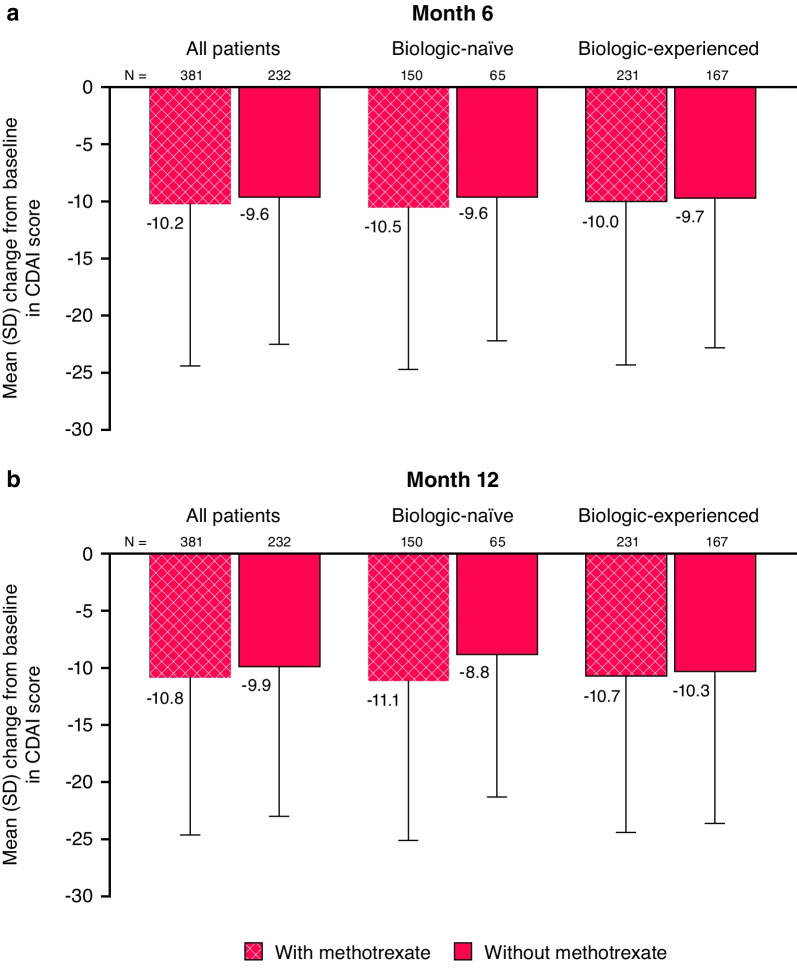
Mean change from baseline at Month 6 (**a**) and Month 12 (**b**) in CDAI score for IV golimumab with and without methotrexate (last observation carried forward for missing data) in biologic-naïve and biologic-experienced patients. *CDAI* Clinical Disease Activity Index, *IV* intravenous, *SD* standard deviation

In the current post hoc analysis of all enrolled golimumab patients with  ≥1 post-baseline CDAI score, the mean ± SD changes from baseline in CDAI score (imputation rule-based analysis) at Month 6 were − 10.2 ± 14.2 and − 9.6 ± 12.9 in the golimumab + MTX (N = 381) and golimumab without MTX (N = 232) groups, respectively (Fig. 1). Corresponding mean ± SD data at Month 12 were − 10.8 ± 13.8 and − 9.9 ± 13.1, respectively. IPTW mean changes in the  golimumab + MTX and golimumab without MTX groups, respectively, were − 10.6 and − 9.0 at Month 6 (LS mean difference: − 1.6 [95% CI − 3.60, 0.37]) and − 11.1 and − 9.2 at Month 12 (LS mean difference − 1.9 [95% CI − 3.89, 0.06]). Additionally, no apparent differences were observed for biologic-naïve and biologic-experienced patients, regardless of concomitant MTX usage. Similar trends were seen when using observed data (Additional file 1: Figure S1).

**Fig. 2:**
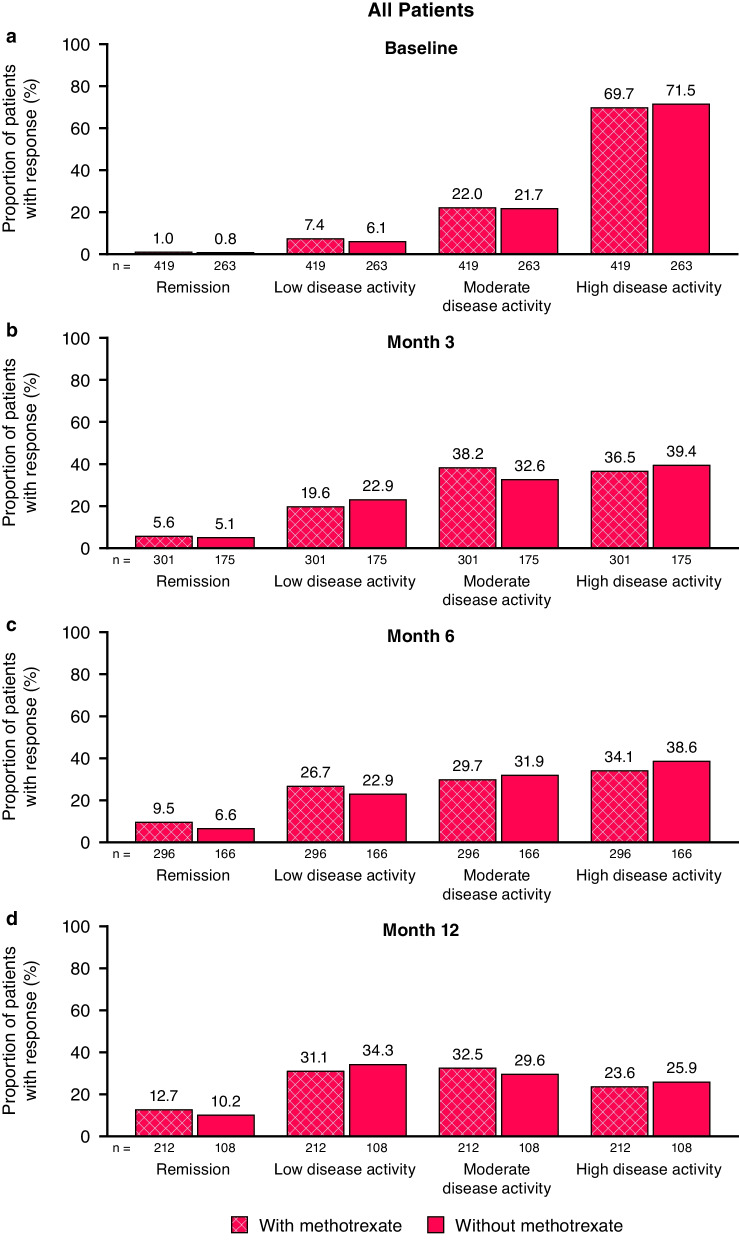
Proportion of all patients in remission (CDAI ≤ 2.8) and with low disease activity (CDAI > 2.8 to ≤ 10), moderate disease activity (CDAI > 10 to ≤ 22), and high disease activity (CDAI > 22) at baseline (**a**) and Months 3 (**b**), 6 (**c**), and 12 (**d**) for IV golimumab with and without methotrexate. *CDAI* Clinical Disease Activity Index, *IV* intravenous

At baseline, the proportions of patients in each CDAI disease activity category were similar between the golimumab + MTX and golimumab without MTX groups, with 22.0% and 21.7%, respectively, having moderate disease activity (CDAI > 10 to ≤ 22), and 69.7% and 71.5%, respectively, displaying high disease activity (> 22) (Fig. 2a). Response rates for achieving remission (CDAI ≤ 2.8) or low disease activity (CDAI > 2.8 to ≤ 10) were similar between the golimumab + MTX and golimumab without MTX groups over time, with 10% to 13% achieving remission and 31% to 34% achieving low disease activity at Month 12 (Fig. 2b–d).

The proportions of patients in each CDAI disease activity category at baseline were generally similar within the treatment groups for biologic-naïve and biologic-experienced patients. However, response rates for achieving remission or low disease activity at Months 3, 6, and 12 tended to be numerically higher in biologic-naïve patients compared with biologic-experienced patients in both the golimumab + MTX and golimumab without MTX groups (Additional file 1: Figures S2 and S3). At Month 12, 35% and 45% of biologic-naïve patients in the golimumab + MTX group and the golimumab without MTX group, respectively, achieved low disease activity compared with 28% and 30% of biologic-experienced patients.

### Safety

At the time of the Week-52 database lock (February 1, 2019), the mean ± SD durations of exposure were 61 ± 42 and 53 ± 42 weeks for the golimumab + MTX group and the golimumab without MTX groups, respectively (Table 2). The total patient-years of follow-up were 575 and 314, respectively.

**Table 2:** Week 52 database lock safety results

	IV golimumab + methotrexate	IV golimumab without methotrexate
Patients, N	420	265
Mean duration of follow-up (weeks)	71.4	61.9
Mean duration of exposure (weeks)	61.4	52.9
Median (IQR) duration of exposure (weeks)	57 (21, 97)	44 (12, 86)
Total patient-years of follow up	574.6	314.3
Patients with ≥ 1 AE	233 (55.5)	146 (55.1)
* Events/100 PY (95% CI)*^*1*^	*155.6 (145.6, 166.1)*	*191.2 (176.2, 207.1)*
Patients with ≥ 1 infection and infestation	121 (28.8)	77 (29.1)
Upper respiratory tract infection	26 (6.2)	11 (4.2)
Urinary tract infection	24 (5.7)	10 (3.8)
Sinusitis	16 (3.8)	20 (7.5)
Bronchitis	17 (4.0)	11 (4.2)
* Events/100 PY (95% CI)*	*37.1 (32.3, 42.4)*	*50.0 (42.4, 58.4)*
Patients with ≥ 1 infusion reaction	12 (2.9)	15 (5.7)
* Events/100 PY (95% CI)*	*2.1 (1.1, 3.6)*	*5.1 (2.9, 8.3)*
Patients with ≥ 1 SAE	50 (11.9)	33 (12.5)
* Events/100 PY (95% CI)*	*14.1 (11.2, 17.5)*	*17.8 (13.5, 23.1)*
AEs of special interest		
Serious infections	12 (2.9)	13 (4.9)
*Events/100 PY(95% CI)*	*2.6 (1.5, 4.3)*	*7.0 (4.4, 10.6)*
Latent tuberculosis	0	1 (0.4)
*Events/100 PY (95% CI)*	*0.0 (0.0, 0.5)*	*0.3* *(0.0, 1.8)*
Opportunistic infections	5 (1.2)	6 (2.3)
*Events/100 PY (95% CI)*	*0.9* *(0.3, 2.0)*	*2.6 (1.1, 5.0)*
Malignancies^2^	15 (3.6)	3 (1.1)
*Events/100 PY (95% CI)*	*3.0 (1.7, 4.7)*	*1.0* *(0.2, 2.8)*
Deaths^3^	7 (1.7)	2 (0.1)
*Events/100 PY (95% CI)*	*1.2 (0.5, 2.5)*	*0.6* *(0.1, 2.3)*

Data presented as n (%) unless otherwise noted

*AE* adverse event, *CI* confidence interval, *IQR* interquartile range, *IV* intravenous, *MTX* methotrexate, *SAE* serious adverse event, *PY* patient-years

^1^Incidence rates could include multiple AEs per patient

^2^Among 15 patients with a total of 17 events in the golimumab + MTX group: squamous cell carcinoma (n = 2; 1 patient had 2 events), lymphoma (n = 1), melanoma (n = 1), ovarian cancer (n = 1), and vulvar cancer (n = 1) occurred in biologic-naïve patients; basal cell carcinoma (n = 2), squamous cell carcinoma (n = 3; 1 patient had 2 events), breast cancer (n = 1), lung adenocarcinoma (n = 1), melanoma (n = 1), and stage IV lung cancer (n = 1) occurred in biologic-experienced patients. Among 3 patients in the golimumab without MTX group, squamous cell carcinoma occurred in a biologic-naïve patient, and basal cell carcinoma (n = 1) and Non-Hodgkin's lymphoma (n = 1) occurred in the biologic-experienced patients

^3^Of the 9 patients who died, 7 received golimumab + MTX (one each due to congestive heart failure [biologic-naïve], acute myocardial infarction, vulvar cancer [biologic-naïve], acute respiratory failure [biologic-naïve], septic shock, stage IV lung cancer, and unknown reason), and 2 received golimumab without MTX (one each congestive heart failure [biologic-naïve] and motor vehicle accident)

Approximately 55% of patients in both groups reported ≥ 1 AE (Table 2). AEs in the infections and infestations system organ class were the most common type, occurring in approximately 29% of patients regardless of concomitant MTX use. Common infections reported in both treatment groups were upper respiratory tract infection, urinary tract infection, sinusitis, and bronchitis. The frequency and types of AEs were similar among biologic-naïve and biologic-experienced patients in both the golimumab + MTX and golimumab without MTX groups (data not shown). The incidence of AEs/100PY (95% CI) was 155.6 (145.6, 166.1) in the golimumab + MTX group and 191.2 (176.2, 207.1) in the golimumab without MTX group.

Overall, 27 golimumab-treated patients had an infusion reaction (12 patients in the golimumab + MTX and 15 patients in the golimumab without MTX groups) (Table 2); the incidence of infusion reactions/100PY (95% CI) was 2.1 (1.1, 3.6) in the golimumab + MTX group and 5.1 (2.9, 8.3) in the golimumab without MTX group. The majority of infusion reactions were of mild intensity; none was considered serious. Among the 27 patients who had an infusion reaction, 7 patients (4/420 [1.0%] golimumab + MTX and 3/265 [1.1%] golimumab without MTX) discontinued the study due to an infusion reaction.

A total of 83 golimumab-treated patients had an SAE (Table 2). The incidence of SAEs/100 PY (95% CI) was 14.1 (11.2, 17.5) for golimumab + MTX and 17.8 (13.5, 23.1) for golimumab without MTX (Table 2). No SAEs related to hematologic or liver transaminase elevations were reported, and rates of gastrointestinal SAEs were similar in both the golimumab + MTX and the golimumab without MTX groups (1.4% vs 1.5%).

Serious infections occurred at rates  of 2.6 (1.5, 4.3)/100 PY for golimumab + MTX and 7.0 (4.4, 10.6)/100 PY for golimumab without MTX (Table 2). One (0.4%) patient was diagnosed with latent tuberculosis (golimumab without MTX group). Eleven golimumab-treated patients had an opportunistic infection. The incidence (95% CI) of opportunistic infections/100PY was 0.9 (0.3, 2.0) for golimumab + MTX and 2.6 (1.1, 5.0) for golimumab without MTX. A total of 18 golimumab-treated patients reported 20 malignancies; the incidence (95% CI) of malignancies/100PY was 3.0 (1.7, 4.7) for golimumab + MTX and 1.0 (0.2, 2.8) for golimumab without MTX.

Nine deaths occurred in the golimumab group (7 received golimumab + MTX and 2 received golimumab without MTX) (Table 2). The incidence (95% CI) of deaths/100PY was 1.2 (0.5, 2.5) for golimumab + MTX and 0.6 (0.1, 2.3) for golimumab without MTX.

## Discussion

FDA-approved dosing for IV golimumab in patients with RA is 2 mg/kg infused over 30 min at Weeks 0 and 4, and every 8 weeks thereafter with concomitant MTX [18]. Previous results from the real-world AWARE study indicated that a majority of golimumab doses administered were consistent with the FDA-approved labeling; however, a substantial proportion of these patients did not receive concomitant MTX [20]. Therefore, we evaluated the effectiveness and safety of golimumab in patients who did and did not receive concomitant MTX.

The CDAI is a validated measure of disease activity (range 0–76, with higher scores indicating more severe disease) and is commonly used in clinical practice [21]. In this post hoc analysis of the AWARE study, patients had a mean baseline CDAI score of > 22, indicating high disease activity. Mean changes in CDAI score at Months 6 and 12 were generally similar between the golimumab + MTX and the golimumab without MTX groups when analyzed using LOCF imputation for missing data, IPTW adjustments, and observed data. Additionally, similar changes in CDAI scores were observed with and without concomitant MTX in both biologic-naïve and biologic-experienced patients. Inadequate response was the most common reason for discontinuation of golimumab; however, it should be noted that 65% of patients in the combined golimumab group had received  ≥1 prior TNFi therapy, suggesting that they may have had recalcitrant disease [22].

The Phase 3 GO-FURTHER study of IV golimumab in patients with RA evaluated only the combination of golimumab + MTX, precluding a comparison of golimumab without MTX vs combination therapy in that study population [9]. Golimumab was previously approved as a subcutaneous (SC) injection in combination with MTX for adults with RA [23]. In the Phase 3 studies of SC golimumab in patients with RA (GO-BEFORE [24] and GO-FORWARD [25]), ACR response rates were higher in the SC golimumab + MTX treatment group than in the SC golimumab without MTX group. In contrast, the results of this post hoc analysis of the AWARE study suggest that patients receiving IV golimumab may achieve improvements in RA disease activity either with or without concomitant MTX.

Unlike the FDA-approved indication for both IV and SC golimumab in RA, both IV and SC golimumab are approved as a monotherapy for adults with psoriatic arthritis (PsA) and ankylosing spondylitis (AS). The Phase 3 studies of IV (GO-VIBRANT) [26] and SC (GO-REVEAL) [27] golimumab in patients with PsA permitted concomitant MTX, and approximately 70% and 50% of patients, respectively, received MTX. Both studies reported similar ACR response rates in golimumab-treated patients who did and did not receive concomitant MTX [26, 27]. Concomitant MTX use was also permitted in the studies of IV (GO-ALIVE [28]) and SC (GO-RAISE [29]) golimumab in AS patients; however, few patients in these studies (14% and 21%, respectively) received concomitant MTX; therefore, similar analyses were not conducted.

Management of patients with RA is complex, with 3–20% of patients experiencing difficult-to-treat disease [30, 31]. Recently, a EULAR taskforce identified several characteristics of patients with difficult-to-treat RA: treatment failure history (i.e., failure of ≥ 2 biological DMARDs/tsDMARDs [with different mechanisms of action] after failing csDMARD therapy [unless contraindicated]); presence of active/progressive disease; and the perception by the clinician or patient that the management of the signs and/or symptoms of RA are problematic [32, 33]. In addition, comorbidities that may limit treatment options [34] and some patient characteristics (older age and obesity) have been associated with difficult-to-treat RA [31, 34, 35]. Although the AWARE study did not systematically identify study participants as having difficult-to-treat RA, golimumab-treated patients in AWARE were, on average, older than 60 years (ranging up to 89 years), and nearly 75% were overweight or obese. Furthermore, nearly half of AWARE participants in this post hoc analysis reported at least one comorbidity, with substantial proportions having hyperlipidemia (28%) and diabetes mellitus (15%). Mean baseline CDAI scores were consistent with highly active disease. In addition, nearly two-thirds of golimumab-treated patients were biologic-experienced, and of these, approximately half had received ≥ 2 prior biologic therapies. The totality of these results suggest that many patients enrolled in AWARE may represent difficult-to-treat RA.

Overall, the frequencies, types of AEs, and exposure-adjusted rates through approximately 1 year in these post hoc analyses were consistent with the known safety profile of golimumab [18]. Importantly, SAEs were uncommon (< 13% of patients and exposure-adjusted rate of 14.1 to 17.8/100 PY) regardless of concomitant MTX administration. In a recent pooled safety analysis of IV golimumab across the three aforementioned Phase 3 studies of patients with RA, PsA, and AS (N = 1248), concomitant MTX use was associated with a higher occurrence of elevated alanine transaminases (from ≥ 3 to < 5 times the upper limit of normal) compared with golimumab without MTX [36]. This is consistent with previous reports of hepatic toxicity following MTX therapy [2, 12]. No SAEs related to liver transaminase elevations were reported in AWARE; however, it should be noted that hematologic and blood chemistry evaluations were absent in this real-world study.

The incidence of infusion reactions was numerically higher in patients receiving golimumab without MTX than in those receiving golimumab + MTX. However, in the primary endpoint analysis of AWARE [20], the incidence of infusion reactions among all golimumab-treated patients was approximately five-fold lower vs infliximab (3.6% vs 17.6%, *p* < 0.001, IPTW-adjusted). In all clinical trials of infliximab across various indications (with varying use of concomitant MTX), 20% of patients experienced an infusion reaction [19]. Of clinical significance, the frequency of infusion reactions among all golimumab-treated patients in the AWARE study was low (4%) and consistent with rates observed in Phase 3 trials of golimumab in patients with rheumatic diseases [9, 10, 20, 26]. The requirement for concomitant MTX in the Phase 3 GO-FURTHER study precludes a similar analysis of infusion reactions and MTX use [9, 10].

Although immunogenicity assessments were not performed in the AWARE study, the reported incidence of antibodies to golimumab from the pooled Phase 3 IV golimumab trials (RA, PsA, and AS) [36] was assessed using a highly sensitive, drug-tolerant, enzyme immunoassay [37]. Overall, approximately 22% of patients were positive for antibodies to golimumab, and 6% developed neutralizing antibodies [36]. The occurrence of antibodies to golimumab, including neutralizing antibodies, was numerically lower in patients who received concomitant MTX compared with those who received golimumab without MTX [36]. Across the three IV golimumab Phase 3 studies, the incidence of infusion reactions was low regardless of antibodies-to-golimumab status [38–40]. However, these findings must be interpreted with caution due to the relatively small number of patients receiving concomitant MTX in the AS study and the lack of a golimumab without MTX population in the RA study.

These results from AWARE are limited by the open-label nature of the study. Additionally, these post hoc analyses were exploratory, the AWARE study was not powered to compare the effectiveness and safety of golimumab with and without MTX, and no adjustments were made for multiplicity of testing. Nevertheless, the sample size was large (265 patients receiving golimumab without MTX and  420 receiving golimumab + MTX), and mean changes from baseline in CDAI score were similar between the treatment groups. As this was an observational study, the post hoc analyses reported here are also limited by the relatively high discontinuation rate and potential imbalances (e.g., demographic, baseline disease characteristics, and prior/concomitant medications) between the golimumab + MTX and golimumab without MTX groups; however, similar results were seen with both imputed and observed data as well as with the IPTW analysis. In addition, results were not stratified by MTX dose nor by duration of MTX exposure as limited documentation about MTX therapy was collected. As specified in the protocol of this observational study, few restrictions were placed on prior RA therapies, and the use of any concomitant medication during the study was at the discretion of the treating rheumatologist. Exposure to other medications for RA, including MTX, was reported by the patients and investigators and may have been subject to recall bias or inconsistencies (e.g., partial information). Due to the manner in which medication use was recorded, we were not able to distinguish between prior and concomitant use of RA medications other than MTX, which may limit the generalizability of the results. Additionally, the reasons for discontinuation of prior or concomitant RA therapies were not collected. Finally, safety data were based on spontaneous reporting and may have led to underreporting of some AEs.

## Conclusions

The totality of the results from the AWARE study provides real-world data on the use of golimumab with or without concomitant MTX in RA patients in the US. IV golimumab patients in this study had, on average, high disease activity and substantial prevalence of comorbidities, and thus may represent difficult-to-treat disease. Findings from these exploratory post hoc analyses of the AWARE study indicate that improvements in CDAI scores were similar for golimumab-treated patients, regardless of concomitant MTX use. Safety findings in patients receiving golimumab with and without concomitant MTX were consistent with the known safety profile of golimumab in patients with RA. The real-world data presented here may assist healthcare providers and patients with RA in making informed treatment decisions.

## Supplementary Information

**Additional file 1**.** Figure S1**. Mean changes from baseline at Month 6 and Month 12 in CDAI score biologic-naïve and biologic-experienced patients receiving IV golimumab with and without methotrexate (observed data).** Figure S2**. Proportions of biologic-naïve patients in remission (CDAI ≤ 2.8) and with low disease activity (CDAI > 2.8 to ≤ 10), moderate disease activity (CDAI > 10 to ≤22), and high disease activity (CDAI > 22) at baseline and Months 3, 6, and 12 by treatment group (IV golimumab+methotrexate and IV golimumab without methotrexate).** Figure S3**. Proportions of biologic-experienced patients in remission (CDAI ≤ 2.8) and with low disease activity (CDAI > 2.8 to ≤ 10), moderate disease activity (CDAI > 10 to ≤ 22), and high disease activity (CDAI > 22) at baseline and Months 3, 6, and 12 by treatment group (IV golimumab+methotrexate and IV golimumab without methotrexate).

## Data Availability

Although these data are not currently publicly available for sharing, requests for sharing can be sent to the Corresponding Author and will be evaluated on an individual basis.

## Abbreviations

AE: Adverse events
ACR: American College of Rheumatology
AS: Ankylosing spondylitis
BMI: Body mass index
CDAI: Clinical disease activity index
CI: Confidence interval
csDMARD: Conventional synthetic disease-modifying anti-rheumatic drug
EULAR: European Alliance of Associations for Rheumatology
FDA: Food and Drug Administration
IPTW: Inverse probably of treatment weighted
IQR: Interquartile range
IV: Intravenous
LOCF: Last observation carried forward
LS: Least square
MTX: Methotrexate
PsA: Psoriatic arthritis
PY: Patient-year
RA: Rheumatoid arthritis
SAE: Serious adverse event
SC: Subcutaneous
SD: Standard deviation
tsDMARD: Targeted synthetic disease-modifying anti-rheumatic drug
TNFi: Tumor necrosis factor inhibitors
US: United States
