# Supplementary material for: Effectiveness and safety of intravenous golimumab with and without concomitant methotrexate in patients with rheumatoid arthritis in the prospective, noninterventional AWARE study
Source: BMC Rheumatol. 2023 Mar 27;7:5. doi: 10.1186/s41927-023-00329-8 (PMC10045110; doi:10.1186/s41927-023-00329-8)

Additional File 1

Figure S1. Mean changes from baseline at Month 6 (a) and Month 12 (b) in CDAI score biologic-naïve and biologic-experienced patients receiving IV golimumab with and without methotrexate (observed data).

CDAI, Clinical Disease Activity Index; IV, intravenous; SD, standard deviation

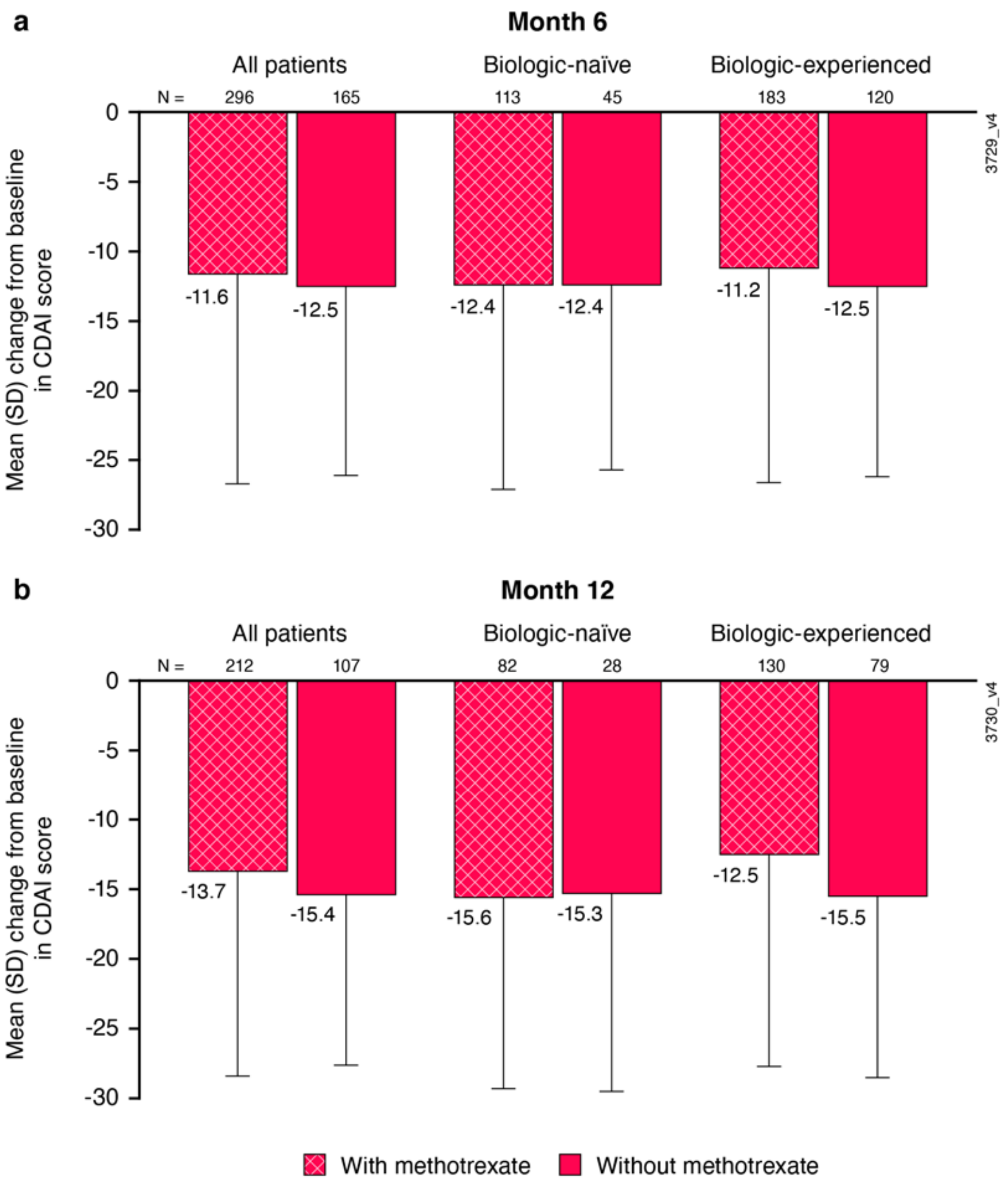

Figure S2. Proportions of biologic-naïve patients in remission (CDAI ≤2.8) and with low disease activity (CDAI >2.8 to ≤10), moderate disease activity (CDAI >10 to ≤22), and high disease activity (CDAI >22) at baseline (a) and Months 3 (b), 6 (c), and 12 (d) by treatment group (IV golimumab+methotrexate and IV golimumab without methotrexate).

CDAI, Clinical Disease Activity Index; IV, intravenous

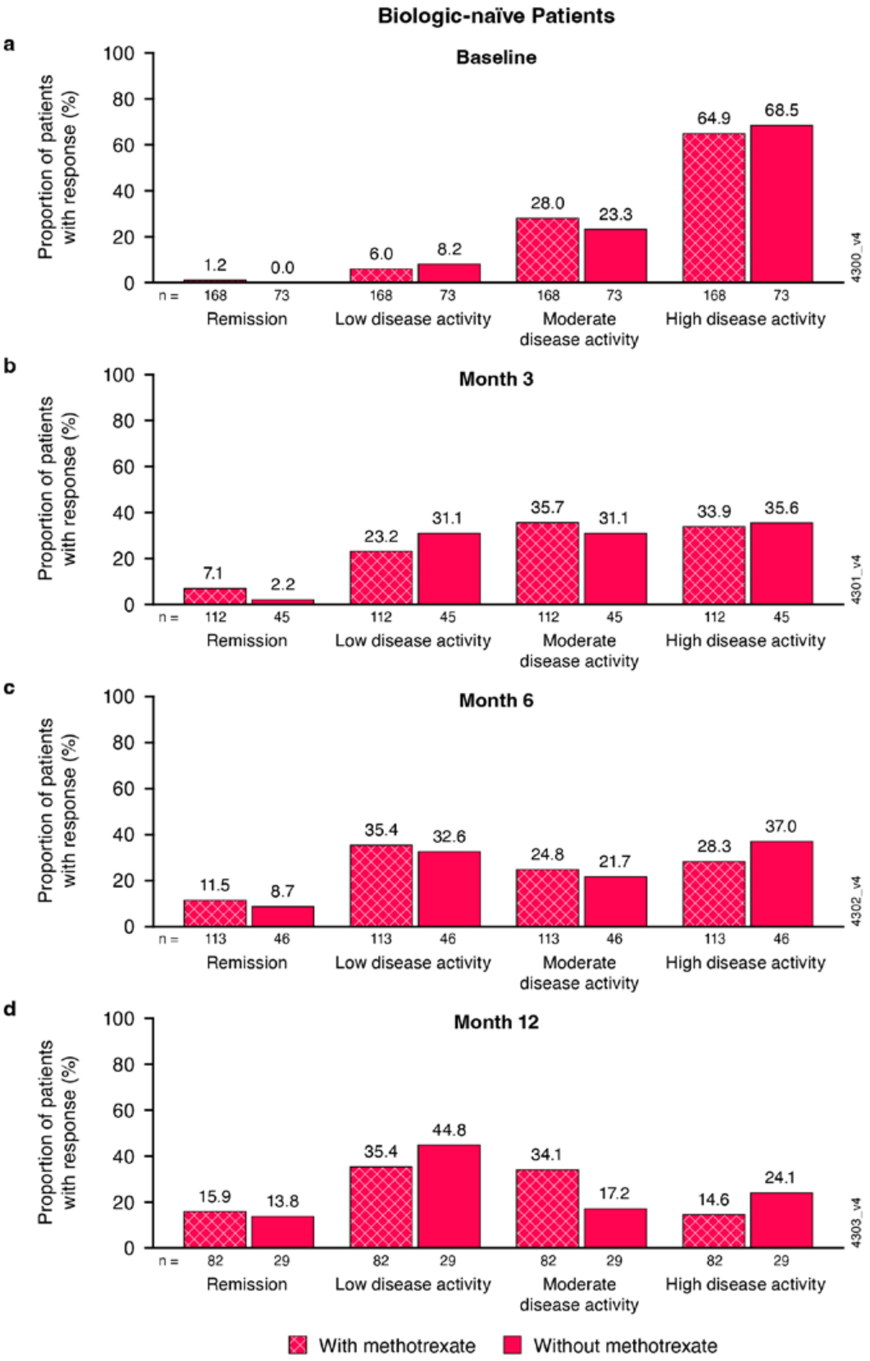

Figure S3. Proportions of biologic-experienced patients in remission (CDAI ≤2.8) and with low disease activity (CDAI >2.8 to ≤10), moderate disease activity (CDAI >10 to ≤22), and high disease activity (CDAI >22) at baseline (a) and Months 3 (b), 6 (c), and 12 (d) by treatment group (IV golimumab+methotrexate and IV golimumab without methotrexate).

CDAI, Clinical Disease Activity Index; IV, intravenous

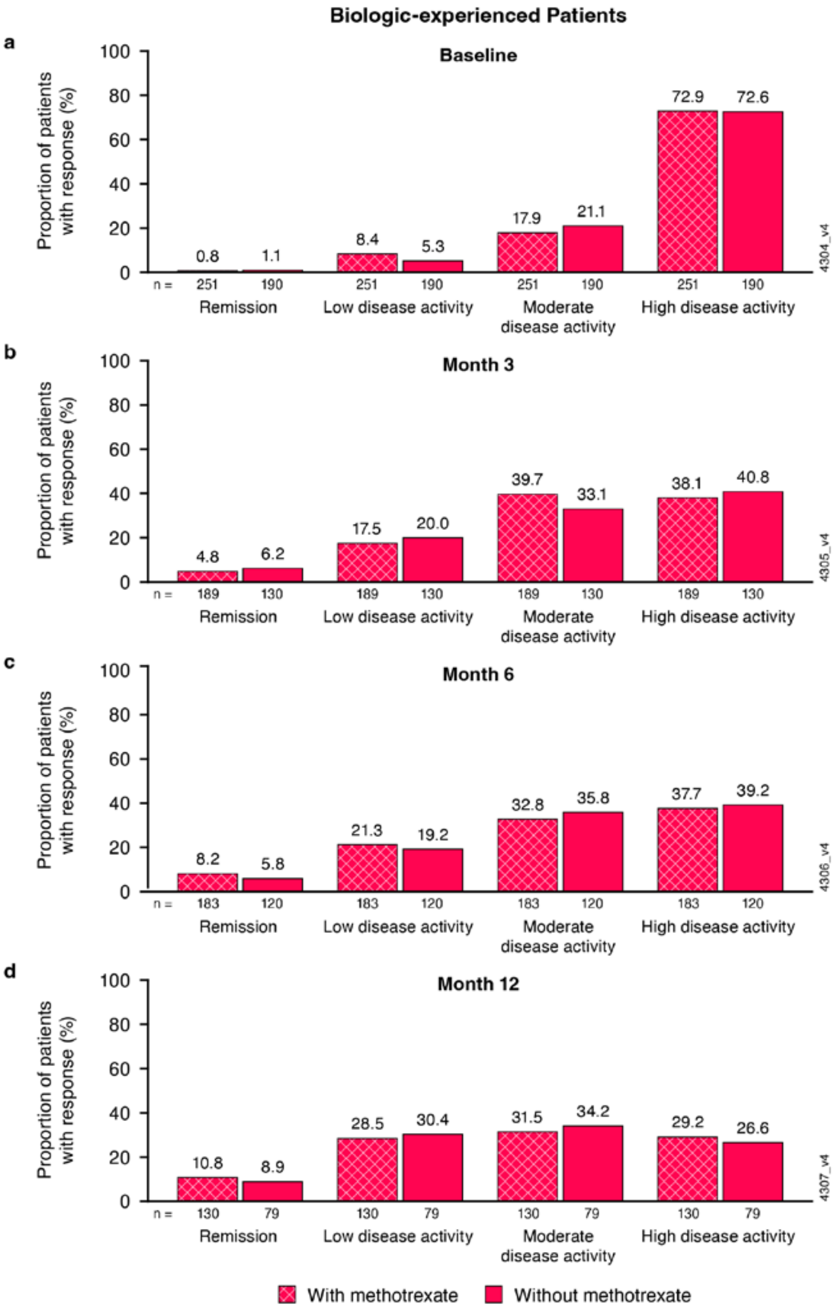

Supplement: Supplementary file 1 — Additional file 1. Figure S1. Mean changes from baseline at Month 6 and Month 12 in CDAI score biologic-naïve and biologic-experienced patients receiving IV golimumab with and without methotrexate (observed data). Figure S2. Proportions of biologic-naïve patients in remission (CDAI ≤ 2.8) and with low disease activity (CDAI > 2.8 to ≤ 10), moderate disease activity (CDAI > 10 to ≤22), and high disease activity (CDAI > 22) at baseline and Months 3, 6, and 12 by treatment group (IV golimumab+methotrexate and IV golimumab without methotrexate). Figure S3. Proportions of biologic-experienced patients in remission (CDAI ≤ 2.8) and with low disease activity (CDAI > 2.8 to ≤ 10), moderate disease activity (CDAI > 10 to ≤ 22), and high disease activity (CDAI > 22) at baseline and Months 3, 6, and 12 by treatment group (IV golimumab+methotrexate and IV golimumab without methotrexate). [file 41927_2023_329_MOESM1_ESM.pdf]
